# Flavonoids in Cancer Metastasis

**DOI:** 10.3390/cancers12061498

**Published:** 2020-06-08

**Authors:** Alena Liskova, Lenka Koklesova, Marek Samec, Karel Smejkal, Samson Mathews Samuel, Elizabeth Varghese, Mariam Abotaleb, Kamil Biringer, Erik Kudela, Jan Danko, Mehdi Shakibaei, Taeg Kyu Kwon, Dietrich Büsselberg, Peter Kubatka

**Affiliations:** 1 Clinic of Obstetrics and Gynecology, Jessenius Faculty of Medicine, Comenius University in Bratislava, 03601 Martin, Slovakia; liskova80@uniba.sk (A.L.); koklesova.lenka@gmail.com (L.K.); marek.samec@gmail.com (M.S.); kamil.biringer@uniba.sk (K.B.); erik.kudela@uniba.sk (E.K.); jan.danko@uniba.sk (J.D.); 2 Department of Natural Drugs, Faculty of Pharmacy, Masaryk University, 61242 Brno, Czech Republic; karel.mejkal@post.cz; 3 Department of Physiology and Biophysics, Weill Cornell Medicine in Qatar, Education City, Qatar Foundation, Doha 24144, Qatar; sms2016@qatar-med.cornell.edu (S.M.S.); elv2007@qatar-med.cornell.edu (E.V.); mariam.abotaleb@aucegypt.edu (M.A.); 4 Musculoskeletal Research Group and Tumour Biology, Chair of Vegetative Anatomy, Institute of Anatomy, Faculty of Medicine, Ludwig-Maximilian-University Munich, D-80336 Munich, Germany; mehdi.shakibaei@med.uni-muenchen.de; 5 Department of Immunology and School of Medicine, Keimyung University, Dalseo-Gu, Daegu 42601, Korea; kwontk@dsmc.or.kr; 6 Department of Medical Biology, Jessenius Faculty of Medicine, Comenius University in Bratislava, 03601 Martin, Slovakia

**Keywords:** cancer, flavonoids, metastasis, phytochemicals

## Abstract

Metastasis represents a serious complication in the treatment of cancer. Flavonoids are plant secondary metabolites exerting various health beneficiary effects. The effects of flavonoids against cancer are associated not only with early stages of the cancer process, but also with cancer progression and spread into distant sites. Flavonoids showed potent anti-cancer effects against various cancer models in vitro and in vivo, mediated via regulation of key signaling pathways involved in the migration and invasion of cancer cells and metastatic progression, including key regulators of epithelial-mesenchymal transition or regulatory molecules such as MMPs, uPA/uPAR, TGF-β and other contributors of the complex process of metastatic spread. Moreover, flavonoids modulated also the expression of genes associated with the progression of cancer and improved inflammatory status, a part of the complex process involved in the development of metastasis. Flavonoids also documented clear potential to improve the anti-cancer effectiveness of conventional chemotherapeutic agents. Most importantly, flavonoids represent environmentally-friendly and cost-effective substances; moreover, a wide spectrum of different flavonoids demonstrated safety and minimal side effects during long-termed administration. In addition, the bioavailability of flavonoids can be improved by their conjugation with metal ions or structural modifications by radiation. In conclusion, anti-cancer effects of flavonoids, targeting all phases of carcinogenesis including metastatic progression, should be implemented into clinical cancer research in order to strengthen their potential use in the future targeted prevention and therapy of cancer in high-risk individuals or patients with aggressive cancer disease with metastatic potential.

## 1. Introduction

The invasion into nearby tissues and the formation of distant metastasis is one of central features of malignancy. Metastasis represents the primary cause of death of cancer patients. Therefore, the understanding of the mechanism metastatic process is an essential step in the identification of therapeutic targets in order to reverse cancer growth and progression [1]. The concept of “soil and seed” determining the establishment of metastasis as a result of an interplay between cancer cells of a primary tumor as well as microenvironment of the pre-metastatic site [2] designates them as important targets of anti-metastatic cancer research [3]. Flavonoids represent a class of secondary plant metabolites exerting potent health beneficiary activities. Flavonoids modulate signaling pathways during not only cancer initiation or promotion, but their effects are significant also in the processes of cancer progression, including invasiveness of surrounding areas and the formation of distant metastasis [4,5,6]. The use of current anti-cancer therapeutics is associated with acquired resistance and reoccurrence of the disease with subsequent spread of metastasis and eventual rise in mortality [7,8,9]. Flavonoids can effectively improve the efficacy of anti-cancer agents [8,10,11]. Therefore, results of the cancer research demonstrating a wide spectrum of anti-cancer effects of flavonoids, mostly in preclinical evaluations, should be necessarily well translated into the clinical cancer treatment research, where there is currently lack of sufficient evidence. Targeted applications of anti-cancer agents based on phytochemicals in high-risk individuals for cancer, as well as personalized therapy of patients with a diagnosed highly-aggressive form of malignancy with a tendency to metastasize, would represent a significant shift in cancer research and later also oncology practice.

### 1.1. Aim of the Study

The review focuses on anti-cancer properties of flavonoids by targeting key steps of cancer progression involving the migration of cancer cells, invasiveness, and formation of metastasis. The core of the review summarizes current knowledge about the efficacy of flavonoids against cancer progression in preclinical and clinical cancer research. Based on the positive results of in vitro and in vivo studies, we emphasize a necessary implementation of flavonoids into clinical research, focusing on a targeted and personalized anti-cancer approach.

### 1.2. Source of the Data

Data were recovered from the biomedical literature by the use of “metastasis” and “flavonoids” or “flavanones” or “flavonols“ or “flavones” or “flavanols” or “isoflavonoids” or “chalcones” or “anthocyanidins” or other associated terms as either a keyword or medical subject heading (MeSH) term in searches of the PubMed bibliographic database. In the special part, focusing on the anti-cancer effects of flavonoids, we emphasize the most recent scientific papers from the years 2017–2020.

## 2. Metastasis: Mechanisms

**Figure 1:**
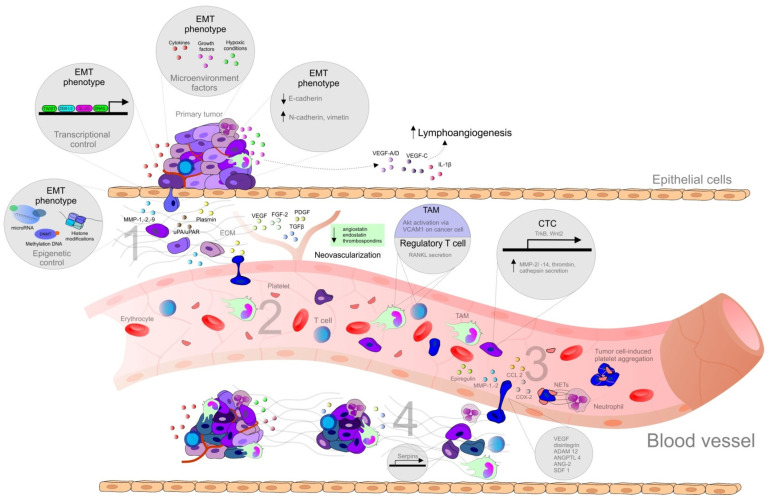
Steps of the metastatic spread from the primary tumor. Abbreviations: ADAM12, metalloproteinase domain-containing protein 12; ANG-2, angiotensin II; ANGPTL4, angiopoietin-like 4; CCL2, CC-chemokine ligand 2; COX-2, cyclooxygenase-2; CTCs, circulating tumor cells; DNMT, DNA methyltransferase; DTCs, disseminated tumor cells; ECM, extracellular matrix; EMT, epithelial-mesenchymal transition; FGF, fibroblast growth factor; IL, interleukin; MAMs, metastasis-associated macrophages; MMPs, matrix metalloproteinase; NET, neutrophil extracellular traps; PDGF, platelet-derived growth factor; RANKL, regulatory T cells producing receptor activator of nuclear factor-κB ligand; SDF1, stromal cell-derived factor 1; Snail, Slug, transcription factors of a snail family; TAM, tumor associated macrophages; TCs, tumor cells; TGFβ, transforming growth factor beta; Twist, basic helix-loop-helix factors; uPA/uPAR, urokinase plasminogen activator/urokinase plasminogen activator receptor; VCAM1, vascular cell adhesion molecule 1; VEGF, vascular endothelial growth factor; ZEB, zinc-finger E-box-binding factors such as the two-handed zinc-finger factors of d-crystallin/E2 box factor (dEF1) family proteins dEF1/ZEB homeobox 1 and Smad-interacting protein 1/ZEB2. *Explanatory notes*: 1. Local invasion: an initiation and maintenance of cancer invasion is facilitated by the regulation of cytoskeletal dynamics in cancer cells and the cell-ECM and cell-cell junctions turnover [14]. EMT that allows cancer cells to accomplish migration and invasion [13] is induced by various stimuli, such as hypoxia, cytokines, and growth factors [15]. EMT is regulated by transcriptional factors (Twist, Snail, Slug, ZEB1, ZEB2) or by epigenetic regulation [15,16]. EMT phenotype is represented by downregulation of E-cadherin and upregulation of N-cadherin (cadherin switch), and vimentin [17]. Remodeling of ECM contributes to cancer progression (clustering of integrins and other receptors → activation of intracellular kinase signaling pathways altering EMT, cancer migration, and invasion) [14]. The detachment from the primary lesions, EMT, migration, and invasion through the basement membrane is followed by degradation of ECM of cancer cells by MMPs (-1, -2, -9) and uPA/uPAR [13]: uPA-uPAR binding → activated uPA catalyzes the conversion of plasminogen to plasmin → degradation of ECM (direct or indirect via activation of MMPs) [18]. Tumor cells escape from immune response and establish a tumor-supportive environment (pre-metastatic niche) in the site of future metastasis [19]. 2. Intravasation and survival in the circulation: the active entry of tumor cells into the circulation is promoted by MMPs or uPA/uPAR [13]. Metastatic process is associated with tumor neovascularization (the secretion of pro-angiogenic stimuli-VEGF, FGF-2, ILs, PlGF, TGF-β, PDGF, angiopoietins etc., and downregulation of anti-angiogenic factors, such as endostatins, angiostatin or thrombospondins) [20], and lymphangiogenesis (VEGF-A/D, VEGF-C, IL-1β, FGF, ECM components, activation of the sympathetic nervous system by chronic stress) [14]. CTCs (overexpressing TrkB or Wnt2) facilitate the avoidance of death stress for epithelial cells from anchorage detachment (anoikis). Other mechanisms allowing the survival of cancer cells in the circulation include the protection against physical shear forces and predation of natural killer cells (secretion of substances, such as thrombin, cathepsin B, MMP-2/-14), the formation of clusters of CTCs and platelets also known as tumor cell-induced platelet aggregation [13,21], and survival signals of TAMs, which activate AKT signaling through vascular cell adhesion molecule 1 (VCAM1) on cancer cells, and regulatory T cells producing receptor activator of nuclear factor-κB ligand (RANKL) [19]. 3. Arrest at distant sites and extravasation: process of CTCs leaving the blood flow (extravasation), [13] facilitating the disruption of vascular junctions and invasion of cancer cells into distant organs, is promoted by epiregulin (EREG), MMPs, COX-2, fascin, and differences in the vascular features of distant organs and primary tumor require additional genes (e.g., *ANGPTL4*) [21]. Metastatic colonization occurs only at certain sites [14] and is facilitated by release of organ-specific chemokines and the expression of appropriate receptors on the surface of the tumor [13], stimulation of neutrophils by cancer cells → neutrophil extracellular NETs supporting metastatic colonization of distant sites. Vascular permeability is modulated by CCL2, VEGF, disintegrin, ADAM12, epiregulin, COX-2, MMP-1, MMP-2, ANG-2, and SDF1 [14]. Cancer cells trapped in an emboli (metastatic site) produce CCL2 to recruit inflammatory monocytes toward metastatic regions, which differentiate into MAMs secreting VEGF [19]. 4. Micrometastasis formation and metastatic colonization: a challenge for just arriving cancer cells include the differences in stromal components, tissue organization, matrix composition, and cytokine environment [21]. Tissue-specific events are required for the survival in sites of various organs [14]. DTCs facilitating the expression of markers of non-cancerous resident cells or mimicking them represent a possible adaptation for survival and allow secondary organ colonization, as was demonstrated in DTCs expressing serpins, that are typically produced by neurons to protect against plasminogen activator-mediated cell death. Moreover, arriving cancer cells are able to subvert resident stromal cells in order to remodel new environments [14].

The development of metastasis is a complex process, which includes the transport of malignant cells from the primary tumor leading to the invasion into target tissues and organs and the development of secondary lesions [12]. In order to understand the cellular and molecular mechanism of metastasis, the concept of the invasion-metastasis cascade was proposed by Isaiah J. Fidler in 2003, and later adapted by S. Valasytan. As is shown in Figure 1, the steps of the development of distant metastasis from primary tumor include local invasion (1), intravasation and survival in the circulation (2), arrest at distant sites and extravasation (3), micrometastasis formation, and metastatic colonization (4) [13].

Importantly, the process of cancer metastasis is a complex series of events, resulting from the interactions between malignant and non-malignant cells, the extrinsic microenvironmental niche including the biomechanics and the biochemistry of the extracellular matrix (ECM), and various secreted factors [14]. Additionally, epigenetic factors, soluble signals (growth factors, cytokines), cell-cell interactions, adhesive signals from ECM components, and ECM mechanical pressure, as well as intratumoral microbiota, are all determinants of metastasis [1]. An ability to destroy matrix barriers, invasion into surrounding tissues, intravasation, extravasation, and metastasis to distant organs require an action of proteolytic systems, such as MMPs [22] regulated by urokinase plasminogen activator/urokinase plasminogen activator receptor (uPA/uPAR), and tissue inhibitors of metalloproteinases (TIMPs) [23]. However, some MMPs influence tumor progression and metastasis formation negatively, and several may also have a dual role, which depends on the type of the cell they are expressed in Reference [24].

While hundreds of thousands of cancer cells intravasate into the blood circulation, only a low number of circulating tumor cells (CTCs) are able to survive and extravasate into distant sites and persist as disseminated tumor cells (DTCs). An even smaller number of DTCs, if they do not become dormant, progress to form metastasis [14]. Additionally, according to Steven Paget’s hypothesis of “seed and soil”, the successful metastasis in the distant tissue is associated both with intrinsic properties of cancer cells (seed), and receptive microenvironment (soil) [2]. Therefore, sites of future metastasis are selectively modified by the primary tumors prior to the CTCs’ arrival. The modification of secondary tissue microenvironments to promote metastatic colonization, a mechanism known as pre-metastatic niche, is a result of complex interactions of factors secreted by tumor and tumor-shed extracellular vesicles, with specific surface marker compositions inducing changes such as induction of vascular leakiness, stroma, and ECM remodeling, and the influence on the immune system [14].

Chronic inflammation is an important player involved in tumor development, as well as in tumor progression, metastasis, and therapy resistance, through alterations of tissue homeostasis and activation of surrounding stromal cells, and recruitment of immune cells [25]. The diversity and plasticity of immune cells allow them to acquire distant phenotypes, either inhibiting or promoting metastasis. Cytokines derived by cancer cells, such as TGFβ or IL-10, contribute to the differentiation of tumor-infiltrating immune cells into a tumor-promoting phenotype, including tumor-associated macrophages (TAMs) and neutrophils (TANs) that can suppress anti-tumor immune response via several mechanisms such as the production of immunosuppressive cytokines or the expression of T cell co-inhibitory molecules. Moreover, aberrantly-produced cytokines or chemokines in the primary tumor induce mobilization and recruitment of myeloid cells from bone marrow, which plays important role in the composition of TME and pre-metastatic organs. Apart from direct production of cytokines by cancer cells, the expression of cytokines is increased in TME [26].

### Epithelial-Mesenchymal Transition

Epithelial-mesenchymal transition (EMT) is a dynamic process of epithelial cell converting into a mesenchymal phenotype involving disruption of cell-cell adhesion as well as cellular polarity, cytoskeleton remodeling, and alteration in cell-matrix adhesion [15], allowing cells to break the basal membrane and invade into adjacent tissues or distant organs [25]. The incomplete state of EMT in cancer cells allows them to possess multiple transitional states and express mixed epithelial and mesenchymal genes, so that such cells can move as clusters and can be more aggressive when compared to cells with complete EMT phenotype [15]. The switch between the individual or collective migration of cells is associated with physical and molecular triggers within the microenvironment [14]. However, the collective cell migration with E-cadherin-dependent cell-cell contacts associated with the epithelial traits has been recently observed in the progression of colorectal, pancreatic, breast cancer, and head and neck squamous cell carcinoma [14]. The downregulation or loss of epithelial E-cadherin leads to the disassembly of adherence junctions and membrane-bound β-catenin translocation to the cell nucleus in which it regulates the transcription of target genes such as *c-myc* or *cyclin D1*. Consequently, the mesenchymal markers including vimentin and neuronal N-cadherin are upregulated while this change from E- to N-cadherin expression, also known as cadherin-switch, enhances the motility of transformed cells [17]. The cleavage of E-cadherin is mediated by MMPs [27]. Cancer cells comprising metastasis are epithelial-like and can be identified as derived from the primary tumor, both morphologically and molecularly. Therefore, cancer cells must reverse mesenchymal phenotype by a reversal EMT, a process known as mesenchymal-epithelial transition (MET). However, the requirement of mesenchymal cancer cells to at least partially reverse to the epithelial state for the metastatic growth is not characterized well [28].

Many cancer types were associated with poor prognosis related to the development of metastasis due to the molecular signatures of primary tumors. Therefore, metastatic gene signature is a property shared by cancer cells of primary tumors [2]. Cancer stem cells (CSC) represent a subpopulation within malignant tumors characterized by self-renewal, differentiation, and tumorigenic potential [29]. Actually, metastasis is suggested to be associated with stem-like properties due to the stem-like gene expression of primary tumors correlating with metastatic and survival outcomes [2]. Besides, increasing evidence suggests that CTCs bears the phenotype of CSCs during the initial process of tumor metastasis [2]. Moreover, the EMT phenotype is also a significant feature of CSCs associated with the metastatic potential [2]. EMT phenotype promotes CSC motility, cancer invasiveness, and metastasis, as well as cancer recurrence and drug resistance. The mesenchymal properties of CSCs are acquired by EMT processes, and then in the target tissues, the MET phenotype allows them to acquire epithelial characteristics [27]. Therefore, targeting the EMT/CSC phenotype may represent a potent therapeutic strategy for metastatic cancer and tumor recurrence [27].

## 3. Targeting Metastatic Cancer

The prevention of the initiation of metastasis in high-risk patients as well as the prevention of additional metastases are included in the concept of cancer therapeutic goals [30]. The major obstacle of the treatment is the biological heterogeneity of the metastasis. The formation of metastasis is the result of a complex series of interrelated steps, while failure in any of these steps can prevent the secondary lesion [3]. The concept of “soil and seed” determines the unique interplay between cancer cells with specific metastatic properties preexisting in the primary tumor and the microenvironment of receptive organs of the future metastasis [3]. However, the phenotype of metastatic tumor differs from the original parent cells because metastasis represents a resistant and invasive subpopulation of primary tumor with additional alterations at the genetic or epigenetic level under prior treatment [31]. Therefore, the targeting of tumor cells, as well as organ microenvironment, can potentially produce more beneficial results [3]. Moreover, as was demonstrated in several cancer types, patients receiving chemotherapy often acquire the resistance, which eventually leads to reoccurrence and metastasis and an increase in mortality [7,8,9]. However, phytochemicals represent a source of anti-cancer agents with great potential in the blockage of metastatic disease [32]. Pleiotropic anticancer efficacy of phytochemicals can be observed in the process of retarding or reversing the metastasis and also in the prevention of invasiveness and metastases [33].

### 3.1. Flavonoids in Cancer Metastasis

**Figure 2:**
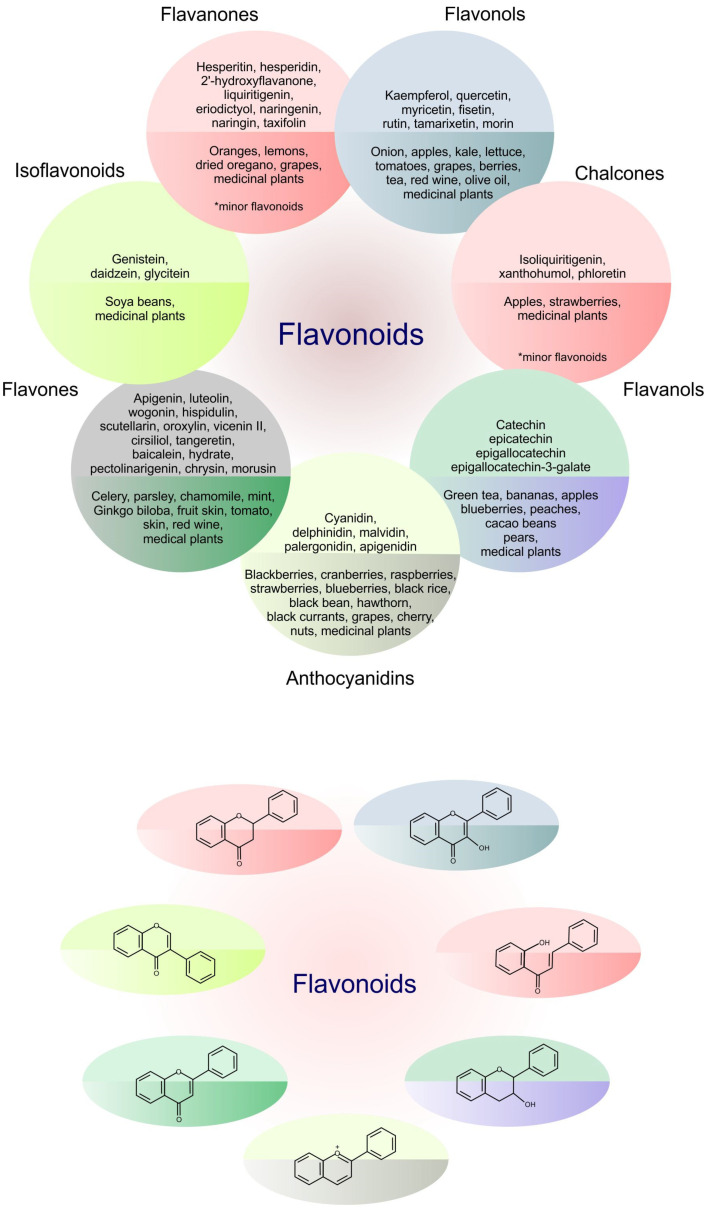
Classification, food sources, and chemical structure of flavonoids [4,10,34,36,37,38,39,40,41,42,43,44,45,46,47,48,49,50,51].

Flavonoids represent an important class of compounds with a phenolic structure, commonly found as glycosides. Flavonoids are widely found in fruit, vegetable, and berry-based beverages. The health-beneficiary effects of flavonoids are associated with their antioxidant, anti-inflammatory, anti-mutagenic, and anti-cancer properties [4,5,6]. The classification of flavonoids is based on their chemical structure, level of oxidation, and pattern of the substitution of ring C (heterocyclic pyrane ring). The substitution of benzene rings (rings A and B) determines the individual compounds within a class [34,35]. Flavonoids can be found in free aglycone form and glycoside-bound form, which is represented by the most commonly-consumed flavonoids in the diet [35]. Figure 2 shows an overview of the classification and food sources of flavonoids, as well as the chemical structure of the subgroups of flavonoids.

#### 3.1.1. Flavonoids in Preclinical Research

The prediction of the efficacy of anticancer agents is the main goal of cancer research. Therefore, pre-clinical cancer models serve as an important tool to screen the anticancer agents with a potential clinical correlation [52].

##### Flavones

**Apigenin**. Apigenin is one of the most common flavonoids found in various vegetables and fruit. Anti-metastatic properties of apigenin, mediated through direct targeting of SPOCK1 and an inactivation of Snail-Slug mediated EMT, were observed in PC-3, PC-3 M and DU145 prostate cancer cells in vitro and suppressed prostate cancer metastasis in vivo in PC-3 M-Luc mice [53]. Similarly, reduction of EMT, migration, and invasion associated with NF-κB/Snail pathway was observed after the treatment with apigenin in HCT-116 and LOVO colon cancer cells in vitro and HCT-116 xenografts in vivo. Apigenin increased the level of E-cadherin and decreased the level of vimentin in HCT-116 cells [54]. In addition, apigenin inhibited the growth of MDA-MB-231 breast cancer xenografts accompanied by reduced levels of IL-6, pSTAT3, pERK, PI3K, pAkt, and N-cadherin. Treatment of MDA-MB-231 cells with apigenin in vitro decreased the migration and invasion, the level of Snail and N-cadherin via inhibition of IL-6. The anti-invasive effect was related to the inhibition of with IL-6 linked signaling pathway, which has an important role in the progression, invasion, and metastasis of breast cancer [55]. Furthermore, apigenin suppressed migration and invasion of melanoma A375 and C8161 cells [56]. Moreover, apigenin impaired the migratory and invasive abilities and exerted antimetastatic effects on melanoma A375, G361 and B16F10 cells via inhibition of STAT3 nuclear organization and transcriptional activity as well as its target genes involved in cell migration and invasion. Apigenin also inhibited melanoma B16F10 cell lung metastasis in an experimental lung metastasis model of C57BL/6 mice [57]. Additionally, apigenin suppressed stem cell-like properties through inhibiting the activity of YAP/TAZ, two main downstream effectors of the Hippo pathway involved in EMT, tumor metastasis, and tumorigenesis in triple-negative breast cancer in which the overexpression of YAP and TAZ correlate with the bioactivity of CSCs, resistance to chemotherapy, and metastasis [58].

**Luteolin**. Flavonoid luteolin decreased the metastasis of highly invasive A431-III squamous carcinoma cells through a reduction of the protein levels of S100 calcium-binding protein A7 (S100A7), phosphorylated p-Src, and pSTAT3. Importantly, S100A7 can activate EMT signaling and promote the metastasis of tumor cells [59]. Interestingly, luteolin inhibited migration and invasion by upregulation of miR-384 and reduced the expression of MMP-2, MMP-3, MMP-9, and MMP-16 in colorectal cancer in HT-29 and SW480 cells. Luteolin also inhibited HT-29 metastasis from the spleen to the liver in nude mice. Moreover, luteolin decreased the *pleiotrophin* (*PTN*) expression, a gene positively related to cancer progression. Results demonstrated that miR-384 directly regulated *PTN* expression in HT-29 and SW480 cells and colorectal cancer tissues [60]. In addition, an inhibition of migration and invasion of A375 malignant melanoma cells and reduction of tumor growth in A375 cells mice xenografts were observed after treatment with luteolin. Luteolin decreased the MMP-2 and MMP-9 expression and increased TIMP-1 and TIMP-2 expression. Inhibition of phosphorylated Akt1 and PI3K suggested that the reduction of MMP-2 and -9 expression was associated with PI3K/AKT pathway [61].

**Wogonin**. Wogonin, one of major flavonoids isolated from *Scutellariae* radix (*Scutellaria baicalensis* L.) reduced lipopolysaccharide (LPS)-induced invasiveness of MDA-MB-231 breast cancer cells through the downregulation of IL-8 and MMP-9 and their association with leukotriene B4 receptor 2 (BLT2)/extracellular signal-regulated kinase (ERK)-linked cascade. Wogonin also decreased the level of 5-lipoxygenase (5-LO) in LPS-stimulated MDA-MB-231 cells and thus inhibited the upregulation of BLT2, which was suggested to contribute to the IL-8 and MMP-9 production. In addition, an administration of wogonin in MDA-MB-231 mice xenografts suppressed LPS-induced MDA-MB-231 cell metastasis [62]. Moreover, wogonin reduced invasion and migration of MHCC97L and PLC/PRF/5 hepatocarcinoma cells through the inhibition of MMP-9 activity [63]. Interestingly, flavonoid wogonoside (a glucuronide of wogonin), a main in vivo metabolite of wogonin, inhibited the tumor invasion and migration in TNF-α-induced MDA-MB-231, MDA-MB-435, and BT-474 cells through the decreased level of TNF-α. Moreover, the decreased level of TRAF2/4 that was observed after treatment with wogonoside led to the inactivation of NF-κB signaling pathway, which subsequently inhibited the expression of Twist1. Additionally, wogonoside reduced the level of MMP-9, MMP-2, vimentin, and CD44v6 in TNF-α-induced MDA-MB-231 and MDA-MB-435 cells [64]. Osteosarcoma is defined as a high-grade malignant bone tumor with the potential for early metastasis. Importantly, wogonin decreased the renewal capacity of human osteosarcoma CSC. Therefore, wogonin showed potential as an agent preventing osteosarcoma CSC metastasis and to preclude the circulating osteosarcoma CSC in the bloodstream [65]. 

**Hispidulin**. Hispidulin is a flavonoid isolated from *Salvia involucrate* Cav., a plant traditionally used in oriental medicine. As demonstrated in the tissue of colorectal cancer patients, higher expression of PIM1 was associated with the degree of local invasion and lymph node metastasis. Hispidulin inhibited growth and metastasis of HT29 and SW480 colorectal cancer cells in vitro by targeting of PIM1 through inhibition of JAK2/STAT3 signaling by generating reactive oxygen species. Moreover, hispidulin reduced CRC pulmonary metastasis in xenograft animal model (Balb/c mice injected with HT29 cells) [66]. Additionally, hispidulin suppressed the migration and invasiveness of hepatocelular carcinoma cell lines SMMC7721 and Bel7402 through inhibition of MMP-2, MMP-9 as well as induction of TIMP-3 expression and inhibited lung metastasis in Bel7402 xenograft in vivo [67].

**Pectolinarigenin.** Pectolinarigenin, a flavonoid present in *Cirsium chanroenicum* (Nakai) Nakai, exhibited anti-migratory and anti-invasive properties in CT26 and HCT116 colorectal carcinoma cells through downregulation of MMP-9 and phosphorylated STAT3. Moreover, pectolinarigenin inhibited abdominal metastasis in the metastatic model of murine colorectal cancer (CT26 cells injected into BALB/c mice) [68]. Similarly, intraperitoneal administration of pectolinarigenin inhibited breast cancer metastasis into lungs in Balb/c mice injected with 4T1 mouse breast cancer cells and suppressed migration and invasion of MCF-7, MDA-MB-231, and 4T1 breast cancer cells in vitro mediated by an inhibition of MMP-2, MMP-9, and p-STAT3 expression and upregulation of TIMP-2 [69].

**Other Flavones.** Scutellarin, a flavonoid isolated from *Scutellaria barbata* D. Don and *Erigeron breviscapus* (Vaniot) Hand.-Mazz., inhibited hypoxia-induced migration and invasion of T24 and UMUC3 bladder cancer cells in vitro, as well as bladder cancer metastasis in vivo. Additionally, scutellarin inhibited EMT induced by hypoxia in both bladder cancer cells in which the PI3K/AKT and MAPK pathways were included [47].

Moreover, long-term exposure to oroxylin A, a flavonoid extracted from *Scutellaria radix*, inhibited metastasis in oral squamous cell carcinoma cells by suppression of CCL2 and a decrease of its downstream targets, including p-ERK1/2, NFκB, MMP2, and MMP9. Despite CCL2, several other cell migration-associated genes such as *LCN2, ID-1, MDK, S100A9* significantly decreased after long-term exposure to oroxylin A. Moreover, oroxylin A inhibited oral squamous cell carcinoma metastasis in vivo [70].

Vicenin II, a 6,8-di-C-glucoside of apigenin, extracted for example from *Dendrobium officinale* Kimura et Migo, suppressed TGF-β1-induced EMT in A549 and H1299 lung adenocarcinoma cells via the deactivation of TGF-β/Smad and PI3K/Akt/mTOR signaling pathways [71].

Cirsiliol, isolated from *Centaurea jacea* L., inhibited MMP-9 and PI3K/AKT/NF-κB signaling pathways in B16F10 metastatic melanoma cells, leading to the modulation of E-cadherin, N-cadherin, Snail and Twist and therefore suppression of EMT [50].

##### Flavonols

**Quercetin**. Quercetin is one of the most widely distributed and studied flavonoids found in various food sources of plant origin [72]. Antimetastatic properties of quercetin were observed in BGC823 and AGS gastric cancer cells via an interruption of uPA/uPAR system, which plays an important role in the cancer metastasis by modulation of NF-κB, PKC-δ, ERK1/2, and AMPKα. Both quercetin treatment and uPAR knockdown decreased MMP-2 and -9 and blocked Pak1-Limk1-cofilin signaling that is associated with the facilitation of cancer metastasis [18]. Moreover, quercetin suppressed metastatic abilities of non-small cell lung cancer (NSCLC) and bone metastasis in an orthotopic A549 xenograft model and suppressed migratory and invasive abilities of A549 and HCC827 cells through the inhibition of Snail-dependent AKT activation and the Snail-independent disintegrin and metalloproteinase (ADAM) 9 expression pathway [73]. Similarly, quercetin inhibited EMT induced by TGF-β1 via the Twist1 suppression and regulation of E-cadherin expression in the SW480 human colorectal adenocarcinoma cell line [74]. Additionally, the suppression of the HGF/c-MET signaling pathway by quercetin contributes to its antimetastatic properties in melanoma A2058 and A375 cells [75]. Quercetin also inhibited STAT3 transcription activity and target genes that are involved in cell growth, migration, and invasion in melanoma A375 and A2058 cells and inhibited murine B16F10 cells lung metastasis in vivo [76]. Moreover, quercetin exerted significant cytotoxic effects against CD44+/CD133+ prostate cancer stem cells. The knockdown of growth factor midkine enhanced the inhibitory efficacy of quercetin on CD44+/CD133+ migration and spheroid formation. Therefore, quercetin and silencing of midkine may represent a strategy to target CSCs that are associated with cancer relapse, migration, and drug resistance [77].

**Myricetin**. Myricetin suppressed lung metastases in the 4T1 mouse model in vivo as well as migration, invasion, and metastasis in the MDA-Mb-231Br breast cancer cell line in vitro by suppression of MMP-2/-9 protein expression as well as expression of ST6GALNAC5, which is specifically expressed in brain metastatic cell lines and upregulated in brain metastasis patients [78]. Similarly, anti-metastatic properties of myricetin against cholangiocarcinoma KKU-100 cells were mediated partly through suppression of STAT3 pathway. A significant abolishment of downstream genes of STAT3 including intercellular adhesion molecule-1 (*ICAM-1*), *MMP-9*, inducible nitric oxide synthase (*iNOS*), and *COX-2* was also observed after the myricetin treatment [79]. Moreover, myricetin inhibited migration, invasion, and EMT and lung and bone metastasis in PC3 xenograft mouse model. The mechanism underlying tumor repressive properties of myricetin is partly explained by the inhibition of PIM1 and disruption of PIM1/CXCR4 interaction. Importantly, CXCR4 is involved in the process of directing metastatic cancer cells to organs expressing CXCL12 and also supports the growth of cancer cells in distant metastasis [80].

**Kaempferol.** Kaempferol suppressed the invasion and migration of 786-O renal cancer cells via the reduction of MMP-2 protein level and activity, and this effect was associated with the downregulation of AKT phosphorylation and focal adhesion kinase (FAK). Moreover, kaempferol inhibited metastasis of 786-O cells into the lungs by 87.4% in the SCID mice model [81]. In addition, kaempferol suppressed migration and invasion of ARPE-19 human retinal pigment epithelial cells via the reduction of protein expression and activity of MMP-2 mediated by increased levels of phosphorylated extracellular signal-regulated kinases 1/2 ERK1/2 [82].

**Fisetin**. Fisetin suppressed the growth and metastasis of triple-negative breast cancer cell lines MDA-MB-231 and BT549 through EMT reversion via suppression of phosphoinositol 3-kinase (PI3K)-Akt-GSK-3β signaling pathway. Moreover, as was demonstrated in in vivo metastatic breast cancer xenograft model, fisetin reduced lung metastasis and modulated the changes in the expression of EMT molecules and PTEN/Akt/GSK-3β in a similar way as in the in vitro model [83]. Similarly, fisetin suppressed the migration, invasion, and stem-cell-like phenotype of human non-small cell lung carcinoma A549 and H1299 cells through attenuation of EMT demonstrated via a decease in signaling proteins acting upstream to EMT involved in the maintenance of mesenchymal phenotype [84], and affected MMPs activity and the level of key metastatic proteins in human osteosarcoma U-2 OS cells [23]. Moreover, the expression of YB-1, which promotes EMT, was inhibited by fisetin in prostate cancer cell line LNCaP in vitro and in the xenograft model of nude mice implanted with the advanced prostate cancer cell line NB26 in vivo [85]. Additionally, fisetin inhibited human melanoma cell A375, SK-MEL-28, and RPMI-7951 invasion through the promotion of EMT via targeting MAPK and NFκB signaling [86].

**Morin Hydrate**. Morin hydrate, a flavonoid isolated from *Morus alba* L., inhibited metastatic potential of 12-O-tetradecanoylphorbol-13-acetate (TPA)-treated MCF-7 breast cancer cells via the inhibition of MMPs, uPA, uPAR, and the Akt/GSK-3β/c-Fos pathway [87].

##### Flavanones

**2′-Hydroxyflavanone, Liquiritigenin, Eriodictyol, Naringenin and Taxifolin.** A citrus flavonoid 2′-hydroxyflavanone (2HF) inhibited EMT, the migration and invasion of PC-3, and DU145 prostate cancer cells through Wnt/β-catenin signaling by suppression of GSK-3β phosphorylation, β-catenin expression, and transactivation [88].

Liquiritigenin, a flavonoid extracted from the roots of *Glycyrrhiza uralensis* Fisch, inhibited HCT116 colorectal cancer cells invasion and EMT by reduction of Runt-Related Transcription Factor 2 (Runx2) and inactivation of PI3K/AKT signaling [89].

Eriodictyol concentration-dependently inhibited tumor growth and metastasis via the downregulation of the PI3K/Akt/NF-κB signaling pathway in U87MG and CHG-5 glioma cells [90].

Naringenin inhibited the migration and invasion of GBM 8901 glioblastoma cells through several mechanisms, including the modulation of MMPs, ERK, p38 and EMT markers [91], as well as the invasion of prostate cancer PC-3 cells via the reversal of EMT-associated proteins and an inhibition of uPA activity [92].

Taxifolin [93] dose-dependently inhibited the proliferation, migration, and invasion of MDA-MB-231 triple-negative breast cancer cells, promoted MET, and inhibited lung metastases in the 4T1 xenograft mouse model [94].

##### Flavanols

**EGCG**. EGCG is a major phenolic compound found in green tea, showing potent cancer preventive and therapeutic abilities. EGCG inhibited MIA PaCa-2 and Panc-1 pancreatic cancer migration and invasion in vitro and in vivo through modulation of EMT demonstrated via the prevention of cadherin switch as well as a decrease in TCF8/ZEB1, β-Catenin and vimentin expression. Inhibition of Akt pathway by EGCG was observed in a time-dependent manner and was achieved by suppression of IGFR phosphorylation and induction of Akt degradation [95].

Epidermal growth factor receptor (EGFR) is a central molecule of cancer cell proliferation as well as invasion and metastasis. Importantly, EGCG in combination with a synthetic retinoid X receptor-γ agonist 6-OH-11-O-hydroxyphenanthrene (IIF) reduced the EGFR phosphorylation at Tyr^1068^ in all tested breast cancer cell lines (MCF-7, MCF-7TAM, MDA-MB-231) and reduced p473AKT phosphorylation in MCF-TAM cells. Besides, different markers of invasion and migration such as CD44, EMMPRIN, MMP-2, and -9 were decreased, while the expression of TIMPs increased in all tested cells [96].

##### Isoflavonoids

**Genistein.** Genistein suppressed the metastatic potential of PC-3 prostate cancer cells through the reduction of MMP-2 activity [97]. Its anti-metastatic potential in SW480 colorectal cancer cells was mediated possibly through inhibition of TTTY18/Akt pathway [98]. Moreover, inhibitory effects on ovarian cancer cells SKOV-3 and A2780CP migration and invasion mediated by genistein and daidzein were associated with FAK suppression. Both genistein and daidzein modulated EMT markers (increased E-cadherin, reduced vimentin) in SKOV-3 cells [99]. Elevated levels of prostate stem cell antigen (PSCA) has been observed in more than 80% of prostate cancer tissues and all cases of bone metastatic prostate cancer in patients. Importantly, the treatment with genistein together with other two flavonoids belonging to the class of flavones (luteolin) and flavonols (quercetin) inhibited the expression of PSCA at the mRNA and protein level in prostate cancer DU145 cells [100].

##### Anthocyanidins

**Delphinidin**. Delphinidin exerted anti-metastatic effects in human DLD-1, SW480, and SW620 colorectal cancer cells via the inhibition of integrin/FAK signaling in which miRNA-204-3p, upregulated by delphinidin, plays an essential role. Moreover, delphinidin inhibited lung metastasis of DLD-1 cells in xenograft model [101]. Additionally, delphinidin inhibited EMT-related protein expression in human osteosarcoma (OS) cell line through ERK/p38 MAPK signaling [102], as well as inhibited EGF-induced EMT in Huh7 and PLC/PRF/5 hepatocellular carcinoma cells by an inhibition of EGFR/AKT/ERK signaling [103].

##### Chalcones

**Isoliquiritigenin and Phloretin**. Isoliquiritigenin, a flavonoid extracted from licorice root repressed EMT accompanied by an increase in E-cadherin and a decrease of mesenchymal markers in SKOV-3 and OVCAR5 ovarian cancer cells [104]. Moreover, anti-metastatic effects of isoliquiritigenin in MKN28 gastric cancer cells may be related to the downregulation of PI3K/AKT/mTOR signaling pathway [105]. Phloretin, a dihydrochalcone present in the peel and root skin of fruit and vegetables, suppressed the invasiveness and migration of SiHa human cervical cancer cells via the downregulation of MMP-2, MMP-3, and cathepsin S, as well as reversed the TGF-β1-induced EMT and downregulation of mesenchymal markers, including fibronectin, vimentin, and RhoA. Phloretin also suppressed lung metastasis in SiHa cells injected by tail vein and subcutaneously inoculated in a tumor xenograft model [46].

The overview of flavonoids exerting anti-invasive, anti-migratory, and overall anti-metastatic properties described in the previous section is shown in Table 1.

#### 3.1.2. Bioavailability and Safety of Flavonoids 

The anti-cancer effectiveness of flavonoids promotes the necessary evaluation of their application in cancer preclinical and clinical research. However, the use of flavonoids (and also other phytochemicals) is associated with several complications. 

An extensive metabolization of flavonoids occurs in the small and large intestine, while a large proportion of flavonoids that are unabsorbed in the proximal intestine reach the colon. The colon microbiota functions as a metabolic reactor, playing an important role in the catabolizing of unabsorbed flavonoids into smaller molecules. Consequently, flavonoids undergo phase I metabolism in the epithelium while resultant metabolites are transported into the liver, in which further phase I and II metabolism result in the more-polar compounds that mediate biological effects in the target tissue [106]. However, the structural complexity of the flavonoids within their subclasses contributes to the differences in their bioavailability or bioactivity so that flavonoids that are most abundantly present in our diet do not necessarily represent the ones that reach the target tissue [107]. Most flavonoids undergo sulfation, methylation, and glucuronidation in the small intestine and liver. The metabolites of flavonoids are generally associated with reduced bioactivity in comparison with parent compounds [108]. However, some metabolites were observed to exert stronger physiological functions than their precursors [109]. Moreover, age, sex, genotype, prescribed medication, habitual diet composition, and gut microbiome are suggested to affect the absorption, distribution, metabolism, and elimination of flavonoids, and therefore, their circulating concentrations, elimination, and tissue exposure to flavonoids [106]. Importantly, the intestinal microbiome plays an essential role in the metabolism of flavonoids as it is deeply involved in the production of metabolites [109,110]. Despite the bioactivity of flavonoids evaluated in vitro, the in vivo bioactivity could be the determinant of their bioavailability [108]. Therefore, an increase in the bioavailability of flavonoids is also considered as an interest of current research.

Most flavonoids are considered safe [111]. However, an excessive intake of flavonoids can be associated with adverse effects, including mild gastro-intestinal symptoms, haemolytic anaemia, increased risk of hepatotoxicity [112], toxic flavonoid-drug interactions, contact dermatitis, or estrogenic-related concerns of male reproductive health and breast cancer [111]. The low solubility of flavonoids represents a problem for their medicinal application. Nevertheless, their low solubility and short residence time in the intestine and lower absorption usually exclude humans from suffering acute toxic effects, except on rare the occurrence of an allergy [34]. Nonetheless, the formulation of quercetin based on lecithin significantly improved the in vitro solubility and oral absorption of quercetin, while the new formulation was found to be as safe as unformulated quercetin and well-tolerated with notable side effects [113].

Therefore, the increase in the bioavailability of flavonoids, as well as an evaluation of safety issues associated with their use, is also considered as an interest of current research. Despite numerous biological properties and therapeutic applications of quercetin [72], its bioavailability is still restricted in clinical practice due to its low absorption, extensive metabolism, and rapid elimination. However, the complexation of quercetin with metal ion could promote its health beneficiary properties as well as increase its bioavailability. The anticancer and anti-metastatic properties of quercetin-zinc complex (Q-ZnCPX) were demonstrated via the reduction of migratory abilities and invasiveness of BFTC-905 human bladder cancer cells in vitro by the regulation of *AKT* and *MT1-MMP*, genes associated with tumor progression. The potential therapeutic value of natural biomaterials structurally modified by radiation against cancer is connected with the improvements of their anti-cancer activities and a reduction of the cytotoxicity. Due to the insignificant toxicity of low concentrations of hesperidin to B16BL6 murine melanoma cells, the modification of its structure was performed, and hesperidin modified by gamma irradiation inhibited lung metastasis of B16BL6 cells in C57BL/6 mice. Smart radiotherapy biomaterials (SRBs) have been recently introduced as a system for delivery of cannabinoids to cancer cells to elongate the exposure, and a sustainable delivery of flavonoid derivative from *Cannabis sativa* L. FBL-03G from SRBs was maintained and delayed local and metastatic tumor progression in the animal model of pancreatic cancer.

#### 3.1.3. The Role of Flavonoids in Cancer Chemotherapy

The anticancer therapy faces several barriers including acquired resistance to chemotherapy and consequent reoccurrence of the disease with the potential spreading of metastasis [8]. Nevertheless, flavonoids may represent an appropriate way to improve the efficacy of conventional therapeutics. Docetaxel (DTX), a chemotherapeutic agent used for the therapy of metastatic cancer, reaches a low response rate [11]. Phosphoinositide 3-kinase/protein kinase B (PI3K/Akt) is involved in the decrease of anti-cancer effectiveness of cytotoxic drugs including DTX [114]. However, the response rate of DTX increases when it is combined with other chemotherapeutic agents. It was documented that the combination of DTX with an agent belonging to the class of flavonoids may improve the efficacy of docetaxel and represent a potent alternative of anticancer therapy. Quercetin acts as an AKT inhibitor suppressing metastasis via inhibition of AKT/MMP-9 pathway via the downregulation of MMP-9. The therapeutic efficacy of co-delivered DTX and quercetin in form of dual DTX/Qu-loaded hyaluronic acid (HA)/polylactic-co-glycolic acid-polyethyleneimine nanoparticles (NPs) (PP-HA/NPs) was evaluated in highly metastatic murine 4T1 breast cancer cells in vitro, and in vivo in the model using Balb/c mice injected with the same cells. Eventually, the downregulation of p-AKT and MMP-9 contributing to the inhibition of cell migration and invasion as well as an enhancement of the drug accumulation in the tumor and lungs were observed after the DTX/Qu-loaded PP-HA/NPs administration suggesting a promising potential in the treatment of metastatic breast cancer [11]. Similarly, synergy of EGCG with gemcitabine, a first-line therapy for patients suffering from metastatic pancreatic cancer, suppressed MIA PaCa-2 and Panc-1 pancreatic cancer cell growth, migration, and invasion associated with modulation of EMT markers and inhibition of Akt pathway [95]. Moreover, Li et al. demonstrated that cyanidin could reverse the drug resistance and enhance the efficacy of oxaliplatin on hepatic cellular cancer. Eventually, cyanidin suppressed migration and reversed changes in EMT biomarkers induced by oxaliplatin and increased its sensitivity in hepatic cellular cancer cell lines via PDK1-PI3K/AKT signaling [10].

#### 3.1.4. Implementation of Flavonoids Targeting Metastasis in Clinical Research

Despite rich evidence found in preclinical in vitro and in vivo studies, only a limited number of studies evaluating the role of flavonoids against cancer cells invasion, migration and metastasis are concerned with clinical research.

Genistein exhibited potent anticancer properties in the experimental cancer research [115]. However, only a few studies evaluated the safety and the potential role of dietary supplements enhancing the efficacy of anticancer treatment. Therefore, Pintova et al. evaluated the safety of genistein combined with standard fluoropyrimidine and platinum-based chemotherapy in the treatment of metastatic colorectal cancer in a phase I/II pilot study. After all, genistein added to FOLFOX or FOLFOX-Bevacizumav was demonstrated to be safe and tolerable [116]. In addition, the bioavailability of EGCG was also improved by the use of Greenselect Phytosome (GSP), a lecithin formulation of a caffeine-free green tea catechin extract, in early breast cancer patients receiving 300 mg of GSP, an equivalent to 44.9 mg of EGCG daily for 4 weeks prior to surgery [117]. Furthermore, phase II trial evaluated the effects of genistein administered for one month prior to radical prostatectomy in US men with localized prostate cancer. Consequently, genistein altered the expression of genes associated with cancer cell motility and metastasis, and specifically increased the expression of *BASP1* and decreased the expression of *HCF2*. The role of genes, which were altered in prostate tissue by genistein, in cancer invasion was evaluated in human prostate cancer cells in vitro, demonstrating the cell invasion to be suppressed by *BASP1* and increased by *HCF2* [118].

**Figure 3:**
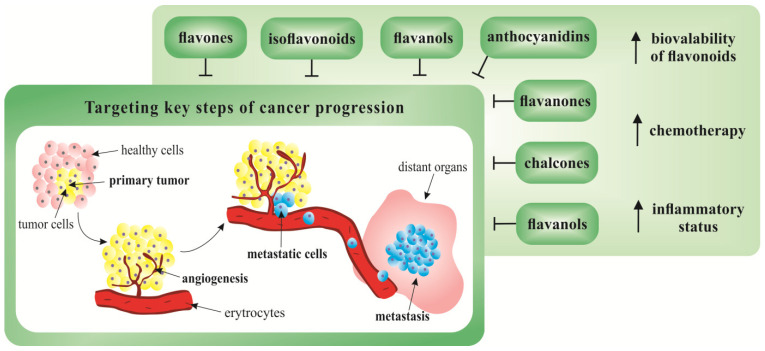
Flavonoids targeting metastasis in current cancer research. Flavonoids exert potent anti-cancer effects targeting key steps of metastatic progression in in vitro and in vivo preclinical cancer studies. Recent clinical cancer research focuses on the bioavailability of flavonoids, as well as their use in the improvements of the efficacy of chemotherapy or other processes associated with metastatic progression, such as the status of chronic inflammation.

Inflammation promotes carcinogenesis, metastasis and therapeutic resistance of colorectal cancer [119,120,121]. Therefore, a double-blinded, randomized placebo-controlled trial evaluated effects of supplementation with fisetin on inflammatory status and MMPs level in 37 CRC patients. The fisetin supplementation, which began a week before chemotherapy and was continuous until the end of the second chemotherapy cycle, decreased plasma levels of IL-8, high sensitivity C-reactive protein (hs-CRP) as well as MMP-7 level. The efficacy of fisetin in the improvement of inflammatory status of colorectal carcinoma patients can be associated with its role of a complementary antitumor agent [119]. The effectiveness of chemotherapy in colorectal cancer patients evaluated as combined with the herbal agent MB-6 composed of fermented soybean extract, green tea extract, *Antrodia camphorata* mycelia, spirulina, grape seed extract, and curcumin extract. Major constituents of MB-6 [122] are known to contain various flavonoids [123,124,125,126,127]. A proof-of-concept clinical study conducted on 72 metastatic colorectal cancer patients randomized to receive leucovorin, 5-fluoroacil, and oxaliplatin in combination with MB-6 or placebo for a period of 16 weeks. After this period, a significantly lower disease progression rate was observed in MB-6 group when compared with the placebo; furthermore, the placebo group showed a significantly higher incidence of adverse events in comparison with the MB-6 group [122]. Figure 3 offers a graphical summary of the use of flavonoids in current cancer research targeting cancer progression.

## 4. Conclusions and Expert Recommendations

The modern clinical approach in the management of metastatic cancer demands the integration of important clinical steps within the multi-professional expertise. It is based on innovative screening programs, genotype stratification, as well as individualized profiling of the patient including multiomic diagnostics [128,129]. Targeting specific cellular signaling is critical for the establishment of cancer metastasis preventing the invasiveness and formation of distant lesions. The beneficial effects of a diet rich in fruits and vegetables are not associated only with the prevention of the initiation of tumorigenesis but also with stopping of cancer progression and increased survival of the patient [130]. As was demonstrated above, flavonoids are able to prevent the onset of the cancer invasiveness in vitro as well as in vivo through the modulation of signaling pathways involved in critical steps of metastatic spread. Moreover, flavonoids were found to be successful anti-cancer agents in highly-aggressive cancer models and the significant efficacy of flavonoids was observed also in the reduction of metastatic spread in several in vivo models.

Regarding the clinical research, flavonoids demonstrated promising results administered in the combination with conventional chemotherapeutics in advanced cancer disease. Nevertheless, the future preclinical and clinical research focused on the role flavonoids in cancer invasiveness should be directed toward the issues important for clinical oncology practice: (1) the definition of precise mechanisms of action, including cellular targets and signaling pathways linked with cancer invasiveness; (2) specifying an effective and well-tolerated doses in patients; (3) progress in the improved bioavailability of flavonoids metabolites by utilizing, for example, nanoparticle carriers; (4) the comparison of the effectiveness of flavonoids in anti-metastatic therapy when applied alone and/or simultaneously with conventional radio-/chemotherapy; (5) the evaluation of anti-metastatic potential (including epigenetic and immunomodulatory mode of action) of highly-specific combination formulas of different flavonoids; (6) analysis of re-sensitizing cancer cells towards conventional chemotherapy and assessing the activities of flavonoids on cancer stem cells survival, affecting the relapse and multidrug resistance, and (7), later, regarding the personalized clinical approach in patients with advanced cancer disease, it will be beneficial better understand to the target mechanisms of flavonoids associated with the individual characteristics, with the aim to develop the most effective combinations of anti-metastatic medications.

The bioavailability and bioactivity of flavonoids are highly influenced by metabolic processes that are affected by many factors, either on the side of individual characteristics of the recipient or the properties related to the flavonoids alone [106,107,108,109,110]. Flavonoids are generally considered safe, but several minor to more severe side effects have been observed with their use [34,111,112,113]. Based on the above, we point to the need for a deeper analysis of the anti-cancer effects of flavonoids in well-defined clinical research, which would also take into account the influence of various factors on the bioavailability of flavonoids, safe doses, and potential toxicity in the individual use of flavonoids or their combination with other agents.

## Figures and Tables

**Table 1:** Flavonoids targeting metastasis in preclinical cancer research.

Natural Compound	Study Design (Dosage of the Tested Flavonoid)	Effects	Mechanism	Reference
Flavones				
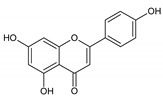 Apigenin	PC-3, PC-3 M, DU145 prostate cancer cells (40 μM); PC-3 M-Luc mice (3 mg/kg)	↓ metastasis ↓ migration ↓ invasion ↓ EMT	↓ SPOCK1 ↓ Snail, ↓ Slug, ↓ vimentin, ↓ N-cadherin ↑ E-cadherin	[53]
	HCT-116 and LOVO colon cancer cells (10 and 20 µM); HCT-116 xenografts (200 and 300 mg/kg)	↓ migration ↓ invasion ↓ EMT	↓ Snail, ↓ vimentin, ↑ E-cadherin, ↓ NF-κB/Snail	[54]
	MDA-MB-231 breast cancer cells (20 and 40 µM); Balb/c mice injected with MDA-MB-231 cells (25 or 50 mg)	↓ invasion ↓ metastasis	↓ IL-6, ↓ pSTAT3, ↓ pERK, ↓ PI3K, ↓ pAkt, ↓ N-cadherin	[55]
	A375 and C8161 cell lines (40 and 80 µM)	↓ migration ↓ invasion	↓ p-AKT and p-mTOR expression level	[56]
	A375, G361 and B16F10 melanoma cells (5, 10, 20 and 40 µM); C57BL/6 mice injected with B16F10 melanoma cells (150 mg/kg)	↓ migration ↓ invasion ↓ metastasis	↓ STAT3 ↓ MMP-9, MMP-2 ↓ VEGF ↓ Twist1	[57]
	MDA-MB-231 and MDA-MB-436 cells (10 and 20 µM)	↓ stem cell-like properties ↓ migration	↓ YAP / TAZ activity	[58]
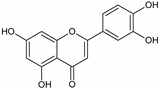 Luteolin	A431-III squamous carcinoma cells (10 and 20 µM)	↓ metastasis	↓ S100A7, ↓ p-Src, ↓ pSTAT3 ↓ Src/STAT3/S100A7	[59]
	HT-29 and SW480 colorectal cancer cells 10 and 50 µM); Balb/c nude mide injected with HT-29 cells (100 mg/kg)	↓ migration ↓ invasion	↓ MMP-2, ↓ MMP-3, ↓ MMP-9, ↓ MMP-16, ↑ miR-384, ↓ PTN	[60]
	A375 human melanoma cells (10, 15, 20 μmol/L); A375 mice xenografts (100 mg/kg)	↓ migration ↓ invasion	↓ MMP-2, ↓ MMP-9, ↑ TIMP-1, ↑ TIMP-2, ↓ pAkt1, ↓ PI3K, ↓ PI3K/AKT	[61]
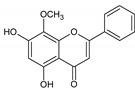 Wogonin	MDA-MB-231 breast cancer cells (10 and 20 µM); MDA-MB-231 mice xenografts (20 μM)	↓ invasion ↓ metastasis	↓ IL-8, ↓ MMP-9, ↓ BLT2, ↓ 5-LO, ↓ BLT2/ERK	[62]
	MHCC97L and PLC/PRF/5 hepatocarcinoma cells (100 μM, 50 μM)	↓ migration ↓ invasion	↓ MMP-9	[63]
	CD133+ CAL72 human osteosarcoma CSC (40–80 μM)	↓ stem cell-like traits	↓ MMP-9	[65]
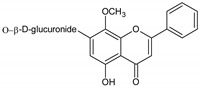 Wogonoside	MDA-MB-231, MDA-MB-435, BT-474 breast cancer cells (100 and 150 μM)	↓ migration ↓ invasion	↓TNF-α-induced metastatic processes, ↓ TRAF2/4, NF-κB inactivation, ↓ Twist1, ↓ MMP-9, ↓ MMP-2, ↓ vimentin, ↓ CD44v6	[64]
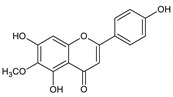 Hispidulin	HT29 and SW480 colorectal cancer cells (10 or 20 μM); xenograft mouse model (20 mg/kg)	↓ metastasis	↓ PIM1 (↓ JAK2/STAT3)	[66]
	SMMC7721 and Bel7402 hepatocelular carcinoma cell lines (10 or 20 μM); xenograft model (20 and 40 mg/kg)	↓ migration ↓ invasiveness ↓ metastasis	↓ MMP-2, ↓ MMP-9	[67]
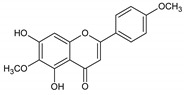 Pectolinarigenin	CT26 and HCT116 colorectal carcinoma cells (2.5, 5, 10, 20 and 40 μM); metastatic murine models (50 mg/kg)	↓ migration ↓ invasion	↓ MMP-2, ↓ p-STAT3	[68]
	MCF-7, MDA-MB-231, 4T1 breast cancer cells (10, 20 and 40 μM); Balb/c mouse model (50 mg/kg)	↓ migration ↓ invasion ↓ metastasis	↓ MMP-2, ↓ MMP-9, ↓ p-STAT3, ↑ TIMP-2	[69]
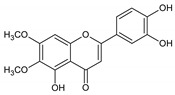 Cirsiliol	B16F10 metastatic melanoma cells (10 µM)	↓ EMT	↓ MMP-9 ↓ PI3K/AKT/NF-κB ↑ E-cadherin, ↓ N-cadherin, ↓ Snail, Twist	[50]
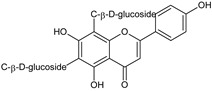 Vicenin II	A549 and H1299 lung adenocarcinoma cells (2.5, 5, and 10 µM)	↓ EMT	TGF-β/Smad and PI3K/Akt/mTOR deactivation	[71]
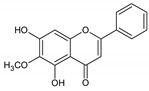 Oroxylin A	Oral squamous cell carcinoma cells (10, 20 μM); mice model (30 mg/mL)	↓ metastasis	↓ CCL2 (↓p-ERK1/2, NFκB, MMP2, MMP9)	[70]
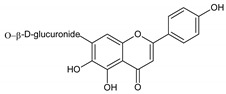 Scutellarin	T24 and UMUC3 bladder cancer cells (30 μM); xenograft mouse model, T24 cells (25)	↓ migration ↓ invasion ↓ EMT		[47]
**Flavonols**				
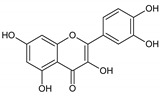 Quercetin	BGC823 and AGS gastric cancer cells (10 μM)	↓ migration ↓ invasiveness	↓ uPA, ↓ uPAR ↓ NF-κb, PKC-δ, and ERK1/2 Activation of AMPKα	[18]
	Non-small cell lung cancer A549 and HCC827 cells (10–50 μM) Orthotopic A549 xenograft model (cells pretreated with 50 μM of quercetin)	↓ migration ↓ invasiveness ↓ metastatic abilities (bone metastasis)	↓ Snail-dependent AKT activation ↓ Snail-independent ADAM9 expression	[73]
	SW480 human colorectal adenocarcinoma cells (25, 50, 100 μM)	↓ EMT	↑ E-cadherin, ↓ Twist1, ↓ vimentin	[74]
	A2058 and A375 melanoma cells (40, 60 μM)	↓ metastasis	↓ HGF/c-MET	[75]
	A2058 and A375 melanoma cells (40, 60, 80 µM); xenograft of human A375 melanoma cells (100 mg/kg); murine B16F10 cell lung metastasis model (100 mg/kg)	↓ migration ↓ invasion ↓ metastasis	↓ STAT3 ↓ Mcl-1, MMP-2, MMP-9, VEGF ↓ murine B16F10 cells lung metastasis	[76]
	CD44+/CD133+ stem cells isolated from PC3 and LNCaP prostate cancer cells (40 μM)	Cytotoxic effects ↓ migration and spheroid formation		[77]
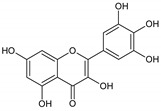 Myricetin	MDA-Mb-231Br breast cancer cells (5, 10 μM); 4T1 mouse model (25 or 50 mg/kg)	↓ migration ↓ invasiveness ↓ metastasis	↓ MMP-2/9, ↓ ST6GALNAC5	[78]
	Cholangiocarcinoma KKU-100 cells (5, 10, 25 μM)	↓ metastasis	↓ STAT3 ↓ ICAM-1, MMP-9, COX-2, iNOS	[79]
	PC3 and DU145 prostate cancer cells (25 or 50 μmol/L); PC3 xenograft mouse model (25 mg/kg)	↓ migration ↓ invasiveness ↓ metastasis ↓ EMT	↓ PIM1 Disruption of PIM1/CXCR interaction	[80]
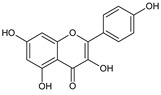 Kaempferol	786-O renal cancer cells (25, 50, 75, 100 μM); SCID mouse model (2 or 10 mg/kg)	↓ migration ↓ invasiveness ↓ lung metastases	↓ MMP-2 (↓ phosphorylation of AKT FAK)	[81]
	ARPE-19 human retinal pigment epithelial cells (25–100 μM)	↓ migration ↓ invasiveness	↓ MMP-2 (↑ ERK1/2)	[82]
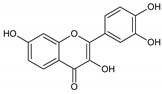 Fisetin	MDA-MB-231, BT549 breast cancer cells (10, 30, 100 μM); metastatic breast cancer xenograft model (100 mg/kg)	↓ growth and metastases	EMT reversion ↓ (PI3K)-Akt-GSK-3β	[83]
	A549 and H1299 non-small cell lung carcinoma cells (10, 25–50 μM)	↓ migration ↓ invasiveness ↓ stem-cell-like properties	↑ E-cadherin (A549) ↑ ZO-1 (H1299) ↓ vimentin, ↓ N-cadherin, ↓ MMP-2 ↓ β-catenin, NF-κB, EGFR, STAT-3 ↓ stem cell signature markers (CD44 and CD133)	[84]
	U-2 OS human osteosarcoma cells (2.5, 5, 10 μM)	↓ cell mobility ↓ migration ↓ invasion	↓ MMP-2, MMP-7, MMP-9, MMP-1 ↓ pEGFR, SOS-1, GRB2, Ras, PKC, p-ERK1/2, p-JNK, p-p-38, VEGF, FAK, RhoA, PI3K, p-AKT, NF-ĸB, uPA ↑ GSK3β, E-cadherin	[23]
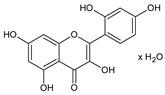 Morin hydrate	LNCaP prostate cancer cells (60 μM); nude mice implanted with prostate cancer cell line NB26 (1 mg/kg / per mice)	↓ EMT	↓ YB-1 phosphorylation ↓ MTA-1 ↓ vimentin ↑ E-cadherin	[85]
	Melanoma cells A375, SK-MEL-28 and RPMI-7951 (5–20 µM)	↓ invasion ↑ MET	↑ E-cadherin ↑ desmoglein ↓ N-cadherin ↓ vimentin ↓ Snail ↓ fibronectin	[86]
	MCF-7 breast cancer cells	↓ metastases	↓ MMP-7, MMP-9 ↓ uPA, uPAR ↓ Akt/GSK-3β/c-Fos pathway	[87]
**Flavanones**				
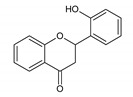 2´-Hydroxyflavanone	PC-3 and DU145 prostate cancer cells (5 and 10 µM)	↓ migration ↓ invasion ↓ EMT	↓ GSK-3β phosphorylation, β-catenin	[88]
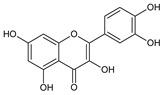 Liquiritigenin	HCT116 colorectal cancer cells (50 μg/mL)	↓ invasion ↓ EMT	↓ Runx2 PI3K/AKT inactivation	[89]
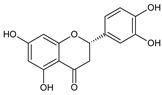 Eriodictyol	U87MG and CHG-5 glioma cells (25, 50, and 100 μM)	↓ growth and metastasis	↓ PI3K/Akt/NF-κB	[90]
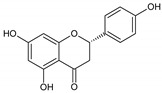 Naringenin	GBM 8901 glioblastoma cells (100, 200, 300 μM)	↓ migration ↓ invasiveness ↓ EMT	↓ MMP-2, MMP-9, ↓ ERK, ↓ p38, ↓ Snail, Slug	[91]
	PC-3 prostate cancer cells (25, 50, 100, 200, and 300 μM)	↓ migration ↓ invasiveness ↓ EMT	↓ uPA ↑ E-cadherin ↓ vimentin, Snail1, Snail2, Twist1	[92]
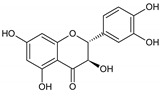 Taxifolin	MDA-MB-231 breast cancer cells (10, 30, 100 μM); 4T1 xenograft mouse model (100 mg/kg)	↓ migration ↓ invasion ↓ metastases ↑ MET	↓ β-catenin	[94]
**Flavanols**				
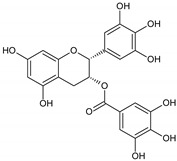 EGCG	MIA PaCa-2 and Panc-1 pancreatic cancer cells (20, 40, 60 µM); C57BL/6J mice injected with KPC cells (10 or 20 mg/kg)	↓ migration ↓ invasion ↓ EMT	↓ TCF8/ZEB1, ↓ β-Catenin, ↓ vimentin, ↓ Akt, ↓ IGFR phosphorylation	[95]
EGCG + 6-OH-11-O-hydroxyphenanthrene (IIF)	MCF-7, MCF-7TAM, MDA-MB-231 breast cancer cells (25 μg/mL)	↓ migration ↓ invasion	↓ EGFR phosphorylation at Tyr^1068^, ↓ p473AKT, ↓ CD44, ↓ EMMPRIN, ↓ MMP-2, ↓ MMP-9, ↑ TIMPs	[96]
**Isoflavonoids**				
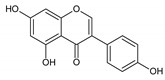 Genistein	PC-3 prostate cancer cells (30, 50, and 70 mM)	↓ metastatic potential	↓ MMP-2	[97]
	SW480 colorectal cancer cells (25, 50, 100 μM)	↓ metastatic potential	↓ TTTY18/Akt pathway	[98]
	SKOV-3 and A2780CP ovarian cancer cells (10 or 50 µM)	↓ migration ↓ invasiveness ↓ EMT	↓ FAK ↑ E-cadherin, ↓ vimentin	[99]
Genistein, luteolin and quercetin	DU145 prostate cancer cells (80 µM each flavonoid)	↓ PSCA		[100]
**Anthocyanidins**				
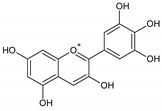 Delphinidin	DLD-1, SW480, SW620 human colorectal cancer cells (50 and 100 μM); xenograft animal model (120.6 ± 5.6 mg and 110.6 ± 0.8 mg)	↓ migration ↓ invasion ↓ metastasis ↓ EMT	↓ integrin αV/β3 ↓ FAK/Src/paxillin signaling ↓ Snail, Slug, Twist, β-catenin, MMP-2 ↑ E-cadherin	[101]
	HOS and U2OS human osteosarcoma cells (75 μM)	↓ EMT	↑ E-cadherin, ↓ N-cadherin ↓ Slug, Snail ↓ ERK and p38 phosphorylation	[102]
	Huh7 and PLC/PRF/5 hepatocellular carcinoma cells (30–100 μM)	↓ EMT	↑ E-cadherin ↓ vimentin, Snail ↓ MMP-2 ↓ EGFR/AKT/ERK	[103]
**Chalcones**				
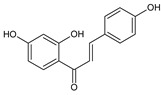 Isoliquiritigenin	SKOV-3 and OVCAR5 ovarian cancer cells (10 μM)	↓ EMT	↑ E-cadherin, ↓ N-cadherin, ↓ vimentin	[104]
	MKN28 gastric cancer cells (20 µM)	↓ metastasis	↓ PI3K/AKT/mTOR	[105]
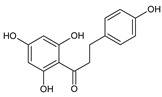 Phloretin	SiHa human cervical cancer cells (100 μM); xenograft model, SiHa cells (20 mg/kg)	↓ migration ↓ invasion ↓ EMT	↓ MMP-2, MMP-3, ↓ cathepsin, ↓ fibronectin, ↓ vimentin, ↓ RhoA	[46]
